# Delivery of primary health care in Malawi

**DOI:** 10.4102/phcfm.v10i1.1799

**Published:** 2018-06-21

**Authors:** Martha T. Makwero

**Affiliations:** 1 Department of Family Medicine, University of Malawi, Malawi

## Abstract

Malawi is a landlocked country with a population of 17 million. The delivery of the health care system is based on primary health care (PHC). The PHC structures are acceptable; however, the system is marked by maldistribution of resources, fragmentation of services and shortage of staff. This hampers the function of the set, well-meaning PHC frameworks. Family medicine offers training and retention of the PHC and rural workforce, harnessing clinical governance and capacity building. Family medicine’s role extends to involve advocacy for the PHC to improve its performance.

## Introduction

Malawi is a landlocked country in southeast Africa. It had an estimated population of 17.4 million people in 2017 with an average annual growth rate of 2.7%, giving an estimated population of 20.4 million people by 2022.^1,2^ Eighty-four percent of Malawi’s population is rural, though an average annual urban population growth rate of 4.2% is expected from 2013 to 2030.^2^ Malawi has a young population, with 64% under the age of 15 years, 18% under the age of 5 years and only 3% above 65 years.^1^ Life expectancy was estimated at 58 years^3^ for both sexes in 2017.^2,4^ The leading causes of disability-adjusted life years (DALYs) include human immunodeficiency virus (HIV) and acquired immune deficiency syndrome (AIDS), respiratory infections, malaria, diarrhoeal diseases and perinatal conditions.^3,4^ Malawi is now having to face a double burden of communicable and non-communicable diseases with 32% and 5.8% of Malawians being hypertensive and diabetic, respectively.^5,6^

Malawi’s economy is largely based on agriculture, fishing and forestry, which contributes 28% of the gross domestic product (GDP).^7^ The country’s GDP per capita in 2015 was estimated at USD381.40 and a growth rate was reported as 2.9% in 2016.^8^ Malawi’s GDP is one of the lowest in sub-Saharan Africa.^9^

## Structure of health care

Health services in Malawi are provided by the public, private for profit (PFP) and private not for profit (PNFP) sectors.^2^ The following governmental departments provide public services: the Ministry of Health (MOH), district, town and city councils, Ministry of Defence and Ministry of Internal Affairs and Public Security (Police and Prisons).^2,10^ These departments work in collaboration with the MOH; however respective funding is planned and disbursed individually.

The PFP sector is small, but growing, and includes an array of private hospitals and clinics that range from group to solo practices. Except for a few larger organisations, most PFP facilities are rudimentary, lacking adequate coordination and regulation. PFP entities are usually solo practices that are staffed by clinical officers or general practitioners. Mostly, PFP sector offers curative services with minimal laboratory support. Another important component of our health care system is the traditional healers and birth attendants who would be considered part of the PFP sector.

The PNFP sector comprises of religious institutions, non-governmental organisations (NGOs), statutory corporations and companies. The major religious providers are organised under the Christian Health Association of Malawi (CHAM) which provides approximately 29% of all health services.^2^ Most PFP and PNFP services charge user fees. Government, through the ministry of health (MOH), enters into different service level agreements with the PNFP sector to provide essential services, such as maternal and child health care, especially in rural and remote areas.^2^

Public sector provision of health care is free and organised into three tiers, primary, secondary and tertiary levels.

There are four tertiary hospitals, which are expected to offer advanced, specialised care, but in reality 70% of services offered at the tertiary level are for conditions that should be treated at primary care or district hospitals. This is partly because of poor gatekeeping mechanisms at the lower tiers of care.^2^

The 26 district hospitals are often rural and act as referral centres for the primary care facilities. District hospitals offer outpatient and inpatient services, surgical procedures like caesarean sections, herniorrhaphy and other emergency life-saving surgeries. District hospital bed capacity can reach up to 300.^2^ Each district hospital has an array of 11 to 40 health centres in its drainage area. Some districts have an intermediary level of community hospitals that are with PFP and PFNP facilities. District hospitals serve populations ranging from 140 000 to 1400 000.^8^

The primary care level includes health centres with their constituent clinics, dispensaries and village clinics. Health centres offer ambulatory and maternity services. Health centres are meant to serve an average population of 10 000 people; however, some urban facilities serve up to 237 000 people. Primary care facilities are meant to link with community-based services, although there is often fragmentation between facility-based curative and community-based preventive activities.

## District health care workforce

The team at district hospitals comprises of one–two doctors, 10–20 clinical officers (also known as clinical associates) and medical assistants who have three and two years of clinical training, respectively.^2^ Other cadres and allied health professionals, such as laboratory technicians and physiotherapists are scantly present at district hospitals and health centres.

The district health management team (DHMT) operates from the district hospital and includes clinical, nursing, environmental and administrative arms. The DHMT plans, monitors and evaluates the district health care activities as a whole.^11^ Despite the said planning, discordance between DHMT’s plans, and priorities with partners’ areas of focus is often observed. Furthermore, programmes’ implementation and financing is verticalised portraying minimal integration, with an unacceptably unbalanced amount of resources allocated for HIV-related activities.

Health centres are staffed by nurses and medical assistants or clinical officers (mid-level practitioners). Nurses deal largely with primary maternal and child health services. Teamwork at health centre level between clinical, nursing and environmental staffs exists, but only to an extent. There exists a fair amount of dichotomy in implementation, monitoring and evaluation of the respective activities.

The community links with the primary care facility are via a team of health surveillance assistants (HSAs), community health workers (CHWs) and traditional healers. HSAs are a community level cadre who received six weeks of initial health preservice training and ideally reside in the community they serve. Each HSA is responsible for about 1500 people, and there are currently about 11 000 HSAs in government service.^7^ HSAs mainly provide health promotion and preventive health care through door-to-door visitations and outreach clinics.^7^ The clinician and nursing roles at the health centre are largely curative, with minimal health promotion and preventive responsibilities. Most of the said roles are taken by environmental staff (HSAs) and CHWs. This dichotomy is seen, for example, during outbreak management or immunisation campaigns, where HSAs take most of the responsibility. CHWs are community volunteers who receive no formal training and act as a conduit for community engagement with the local community leaders and health advisory committees.

In the community, HSAs are seen as ‘doctors’. Their roles extend from sanitation, health promotion and community diagnosis to treatment of minor illnesses, especially in hard to reach areas as part of an integrated management of childhood illnesses programme.^7^ Within the community, HSA’s work through the village leadership and CHWs as a team. With current efforts on decentralisation, efforts are being invested to harness community involvement to collectively contribute towards solutions to improve primary care outcomes.

## Progress on health and patient care

Malawi made significant strides in meeting the millennium development goals for literacy, childhood mortality, HIV and malaria.^4^ Other areas, for example, maternal health, still require innovation and further effort.

Malawi’s efforts to attain the sustainable development goals are seen in its attempts to address the World Health Organization (WHO)s six building blocks of health service delivery namely; human resources for health (HRH), infrastructure, medicines and technology, health financing, health information systems, leadership and governance. For example, Malawi has rolled out its decentralisation policy programme in an attempt to devolve health service governance to facilitate community ownership and participation for delivery of EHP.^11^ The progress of this reform, however, has been thwarted by lack of capacity and resources at the decentralised platforms to carry out their respective duties.^10^

Malawi has shown commitment to universal health coverage by extending its original essential health care package to include non-communicable diseases, and continues to be free to all Malawians within public and most PFNP sectors.^2^ The capability of the primary health care sector to deliver this package needs to be strengthened and extended beyond access and coverage, and also for improvement of quality.

## The contribution of family medicine

Family medicine (FM) is a discipline that aims to train doctors with a versatile skill mix that enables provision of care that is holistic, continuous and coordinated across the life cycle and diverse group of patient conditions, extending care to their families and communities. The FM has aligned itself to the national agenda of strengthening primary health care services delivery and access at district health level and below. Thus, FM operates in the decentralised platforms, harnessing functional links between district hospitals and their primary care facilities, thereby transforming PHC at a number of areas. Some key areas are improving the quality of clinical governance, increased access to better quality of care, advocacy for better allocation of resources for PHC, training and retention of a more skilled rural workforce.^12^ Another contribution is that FM is committed to capacity building of the multidisciplinary teams within PHC as a whole.^12^

## Current state of primary health care

Malawi’s disease burden and analytical profile of key determinants of health, along with its successes and failures, are to a greater extent embedded in the performance of its PHC.^4^ For example, its celebrated achievements in reducing childhood mortality is largely because of investments in the extended immunisation programme, primary and community case management.

Theoretically, Malawi has reasonable PHC structures. However, in practice, the health system is marked by lack of resources, maldistribution of staff and funding between rural and urban settings and across tiers of care. Additionally, demotivated staff, task shifting and lack of good interdisciplinary models of work thwart implementation progress. An example of maldistribution of resources is evidenced in staff deployment where 50% of doctors and nurses are stationed in the four central hospitals.^2^ This highlights lack of equity in deployment policies. Additionally, there are high vacancy rates (up to 80%) across all levels, but more so for senior medical officer positions.^2^ These factors impede the delivery of a ‘would have been good’ primary health care model.

Whilst Malawi strives to base its health service delivery on the principles of PHC with community participation as a central approach in addressing health needs of its people,^7^ in practice PHC delivery remains fragmented and community participation is poorly coordinated.

The current PHC structure with decentralised governance platforms and community involvement seeks to support PHC by expanding access to all levels of care. However, there are other determinants of access like cultural barriers and poor quality of services which need to be tackled from a multisectoral approach.^11^ Some of the barriers to providing quality primary health care have been a lack of resources and capital investments, poor human resource deployment policies, funding models that do not favour PHC prioritisation, as well as poor coordination and integration of services.

Continued government commitment towards PHC is a big investment to health and is commended. It is not clear though to what extent this commitment and prioritisation of PHC is communicated among local and external partners within the health sector. This is evidenced by the substantial resources that are allocated to strengthening vertical programmes that hardly support PHC delivery as an entity. It is well known that health care provision in Malawi is highly dependent on external financing. Local and international partners’ budgetary and service support to districts and health centres ought to be coordinated in pursuit of priority district health goals. This requires high-levelled political commitment and support if we are to improve cost-effectiveness and achieve quality. The Health Sector Strategic Plan (HSSP) II has, therefore, focussed on strengthening governance of the health sector in order to improve efficiency and get the maximum out of existing human, financial and material resources. Family medicine has situated itself at the heart of the decentralised platform to help improve PHC functioning and capacity building.

## Lessons for other countries

Putting infrastructure in place for PHC and improving access is not enough. Countries need to invest in improving the quality of primary care, integrating facility and community-based teams, and strengthening community participation. The multidisciplinary team may also benefit from the inclusion of family physicians.
